# Effectiveness of Nutrition-Specific Interventions for Reducing Child Stunting: A Systematic Review of Evidence

**DOI:** 10.3389/ijph.2026.1609291

**Published:** 2026-04-15

**Authors:** Eman Salim Ahmed Salim, Veni Hadju

**Affiliations:** 1 Department of Immunology, The National Ribat University, Khartoum, Sudan; 2 Faculty of Public Health, Hasanuddin University, Makassar, Indonesia; 3 Department of Nutritional Sciences, Faculty of Public Health, Hasanuddin University, Makassar, Indonesia

**Keywords:** child growth, LNS, maternal nutrition, micronutrients, nutrition-specific intervention

## Abstract

**Objectives:**

This systematic review aimed to evaluate the effectiveness of nutrition-specific interventions in improving child linear growth and reducing stunting during the first 1,000 days of life.

**Methods:**

A systematic review was conducted in accordance with PRISMA 2020 guidelines. Searches were performed in PubMed/MEDLINE, Scopus, Web of Science, and the Cochrane Central Register of Controlled Trials for studies published up to September 2025. Randomized controlled trials, cluster-randomized trials, cohort, and quasi-experimental studies assessing nutrition-specific interventions were included. Due to substantial heterogeneity across studies, findings were synthesized narratively.

**Results:**

Thirteen studies conducted in Asia, Africa, and Latin America were included. Nutrition-specific interventions, particularly lipid-based nutrient supplements, fortified foods, and food-based strategies, were associated with modest improvements in length-for-age z-scores and reductions in stunting prevalence. Larger effects were observed when interventions were initiated early in life and implemented in settings with high baseline stunting and food insecurity.

**Conclusion:**

Nutrition-specific interventions can contribute to improvements in child linear growth, especially when delivered early and sustained during the first 1,000 days. However, effect sizes vary by context, underscoring the importance of integrated and context-sensitive implementation strategies.

## Introduction

Child stunting is still one of the most common signs of long-term malnutrition and a major obstacle to human development [1]. Stunting is when a child’s height-for-age z-score (HAZ) is below −2 standard deviations from the WHO Child Growth Standards [2]. This means that the child has not gotten enough nutrition for a long time and has been sick a lot in the early years of life. In 2022, about 148 million kids under 5 years old around the world were stunted. Most of the burden was in low- and middle-income countries (LMICs), especially in South Asia and sub-Saharan Africa [3]. The effects of stunting go beyond just not growing physically; they also include problems with cognitive development, lower levels of education, and lower productivity in the workplace as an adult [4].

The conceptual framework of child undernutrition delineates insufficient dietary intake and disease as the immediate factors contributing to growth retardation [5]. Nutrition-specific interventions tackle these direct causes by delivering essential nutrients or encouraging optimal feeding practices via health and community delivery platforms [6]. Micronutrient supplementation, lipid-based nutrient supplements (LNS), fortified complementary foods, and the promotion of exclusive and continued breastfeeding are all examples of these kinds of interventions [7].

Evidence from multiple randomized controlled trials (RCTs) and community-based initiatives has shown that prompt nutrition-specific interventions, especially during the “first 1,000 days” from conception to 2 years of age, can markedly enhance linear growth outcomes [8, 9].

Even though there is a lot of evidence, the effects that have been reported are very different depending on the setting and type of intervention. While some trials showed substantial improvements in linear growth, others found little difference or only statistically non-significant benefits [10]. This variability is likely driven by differences in underlying nutritional status, food security, infection burden, and program delivery intensity. On the other hand, a comprehensive aggregation of findings is necessary to make an overall judgment about the effectiveness of nutrition-specific interventions for mitigating stunting in early childhood across populations [11].

Despite the expanding body of literature, important gaps remain in understanding the overall strength and certainty of evidence supporting nutrition-specific strategies. Many studies differ in design, duration, and outcome measurement, and long-term sustainability of growth effects is often insufficiently assessed. A comprehensive evaluation of both effect patterns and methodological quality is therefore essential.

### Objectives

This systematic review aimed to evaluate the effectiveness of nutrition-specific interventions in improving child linear growth and reducing stunting during the first 1,000 days of life, and to assess the consistency, contextual variability, and overall certainty of the available evidence.

## Methods

### Study Design and Reporting Framework

This study is a systematic review designed to synthesize evidence on the effectiveness of nutrition-specific interventions in reducing stunting and improving child growth outcomes. The review was conducted and reported in accordance with the Preferred Reporting Items for Systematic Reviews and Meta-Analyses (PRISMA) 2020 guidelines to ensure methodological transparency, reproducibility, and completeness in the identification, appraisal, selection, and synthesis of the included studies.

### Protocol and Registration

A review protocol was developed prior to the conduct of the literature search to define the review objectives, eligibility criteria, and methodological approach. Although this systematic review was not registered in the PROSPERO database or another publicly accessible registry, this is acknowledged as a limitation of the study. Nevertheless, all methodological steps were carefully defined in advance and applied consistently throughout the review process to minimize selective reporting and enhance methodological transparency.

### Search Strategy

A systematic literature search was performed utilizing PubMed/MEDLINE, Scopus, Web of Science, and the Cochrane Central Register of Controlled Trials (CENTRAL). The search encompassed studies published until September 2025, utilizing combinations of Medical Subject Headings (MeSH) and keywords such as “stunting,” “linear growth,” “height-for-age,” “child,” “nutrition intervention,” “lipid-based nutrient supplements,” “micronutrient powders,” “complementary feeding,” and “breastfeeding promotion.” We also looked through reference lists of relevant reviews and included studies to find more articles that could be used.

### Eligibility Criteria

#### Studies Were Qualified if They

1. Employed randomized controlled trial (RCT), cluster-randomized trial (cRCT), cohort, or quasi-experimental design;
2. Included children under 5 years old or women who were pregnant or breastfeeding (within the first 1,000 days framework).
3. Implemented nutrition-specific interventions as defined by the Lancet Maternal and Child Nutrition Series, including micronutrient supplementation, fortified complementary foods, lipid-based nutrient supplements, or promotion of breastfeeding and appropriate complementary feeding.
4. Reported linear growth metrics, including height-for-age z-score (HAZ), length-for-age z-score (LAZ), or the incidence of stunting (HAZ <−2 SD).

Studies that only looked at nutrition-sensitive or WASH interventions, or that only looked at children with severe acute malnutrition, were not included.

### Study Selection and Data Extraction

Two reviewers independently evaluated titles, abstracts, and full texts based on the inclusion criteria outlined in PRISMA 2020 guidelines. Consensus resolved the discrepancies. We took data from each eligible study and put it into a standard form. Using the PICO approach in the first step of the research. The population in this literature study were pregnant mothers and children under five who received nutritional interventions. Data extraction included information about the study (author, year, country, design), the population, the type of intervention, the comparator, and the main outcomes on linear growth or stunting prevalence.

The final synthesis encompassed 13 qualifying studies, consisting of individual RCTs, cluster-RCTs, and quasi-experimental community programs executed in Asia, Africa, and Latin America.

### Risk of Bias and Certainty of Evidence Assessment

The risk of bias of the included studies was assessed at the study level using criteria appropriate to the respective study designs. For randomized and cluster-randomized controlled trials, key domains evaluated included random sequence generation, allocation concealment, blinding of participants and outcome assessors, completeness of outcome data, and selective outcome reporting. For quasi-experimental and cohort studies, attention was given to comparability of groups, outcome measurement, and potential confounding factors.

Given the diversity of study designs, interventions, and reporting standards, risk of bias was assessed narratively rather than quantitatively.

Risk of bias was assessed independently for each included primary study. Randomized controlled and cluster-randomized trials were evaluated using the Cochrane Risk of Bias 2 (RoB 2) tool, examining domains including the randomization process, allocation concealment, blinding of outcome assessment, incomplete outcome data, and selective reporting.

The quasi-experimental study was assessed using structured appraisal criteria aligned with ROBINS-I principles, focusing on confounding, selection bias, and outcome measurement bias. Cohort follow-up analyses were evaluated descriptively with attention to attrition and measurement bias.

Certainty of evidence for key outcomes was rated qualitatively (High, Moderate, Low) based on study design, overall risk of bias patterns, consistency of findings, and precision of reported effect estimates, in accordance with GRADE principles.

### Data Synthesis and Handling of Heterogeneity

A quantitative meta-analysis was not conducted due to substantial heterogeneity across the included studies. The studies varied considerably in terms of intervention type and composition, dosage and duration, target populations, study designs, outcome definitions, and follow-up periods. Such methodological and clinical diversity limited the comparability of effect estimates and precluded meaningful statistical pooling.

Therefore, a narrative synthesis approach was adopted. Findings were synthesized and compared qualitatively by grouping studies according to intervention categories, timing of exposure within the first 1,000 days of life, and contextual characteristics such as baseline stunting prevalence and food insecurity. This approach allowed for a structured interpretation of patterns, consistencies, and variations in intervention effects across different settings.

## Results

### Study Selection Results

**FIGURE 1:**
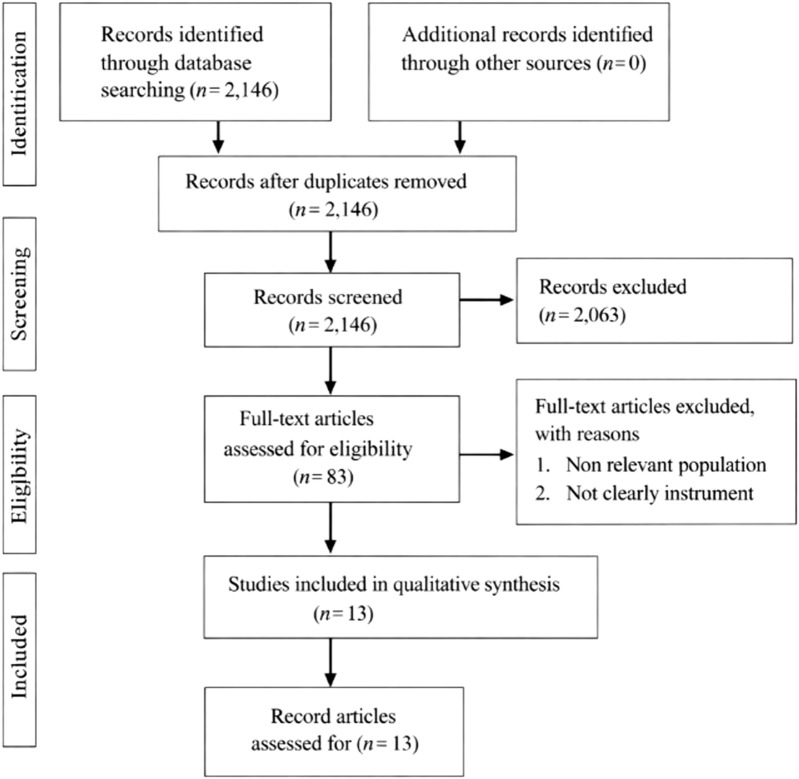
Flow diagram illustrating the study selection process according to the Preferred Reporting Items for Systematic Reviews and Meta-Analyses guidelines (multiple countries, 2008–2024).

The database search identified 2,146 records. After removal of duplicates and screening of titles and abstracts, 83 full-text articles were assessed for eligibility. Of these, 70 articles were excluded for not meeting the inclusion criteria. Thirteen studies were included in the final qualitative synthesis. The study selection process is illustrated in Figure 1 using PRISMA flow diagram.

### Study Characteristics

The included studies were conducted between 2008 and 2024 across Asia, Africa, and Latin America. Most studies (n = 11) were randomized controlled or cluster-randomized trials, while the remainder consisted of one quasi-experimental program evaluation and one cohort follow-up studies of baseline trials.

Sample sizes ranged from approximately 163 to 6,000 participants. All studies evaluated nutrition-specific interventions implemented during the first 1,000 days of life.

The nutrition-specific interventions assessed across studies included lipid-based nutrient supplements (LNS) provided to pregnant women and young children, micronutrient powders (MNPs) and multiple micronutrient (MMN) supplementation, fortified blended or ready-to-use foods (e.g., RUSF, CSB++), food-based interventions such as daily egg supplementation, and integrated programs combining food supplementation with social and behavior change communication (SBCC). The key characteristics and main findings of the included studies are summarized in Table 1.

**TABLE 1:** Summary of included studies on nutrition-specific interventions and stunting outcomes (Asia, Africa, and Latin America, 2008–2024).

No	Author (Year)	Journal	Location	Type of study	Population	Intervention (nutrition-specific)	Comparator	Key findings on stunting (HAZ/LAZ/prevalence)
1	Iannotti et al. [12]	Pediatrics/AJCN	Ecuador (cotopaxi)	Individual RCT	Infants 6–9 months (n ≈ 163)	One egg/day for 6 months (food-based)	Usual diet (no egg)	LAZ MD ≈ +0.63 at endline; stunting ↓ ≈47% vs. control (large effect). Follow-up showed attenuation after 2 years
2	Dewey et al. [13]	American J Clin Nutr (IPD)	Multi-country (pooled LMIC trials)	Individual-participant data meta-analysis (pooled RCTs)	Children ∼6–24 months (pooled)	Small-quantity lipid-based nutrient supplements (SQ-LNS)	No SQ-LNS/usual care	Pooled LAZ MD ≈ +0.14; stunting prevalence ↓ ≈12% (absolute ≈5 ppt); larger benefit in high-burden settings
3	Prado et al. [14]	Maternal & Child Nutrition/AJCN analyses	Multi-country (pooled)	IPD subgroup analysis	Children 6–24 months (pooled)	SQ-LNS	Control	Effect modification: greater SQ-LNS benefit on HAZ/stunting in high-stunting and poorest households
4	Adu-Afarwuah et al. [15]	AJCN (iLiNS-DYAD Ghana)	Ghana	Randomized controlled trial	Pregnant women → infants	Maternal and/or child SQ-LNS (child 20 g/d)	Iron-folic acid (maternal)/usual care	Mixed results: some birth-size increases in subgroups; modest/variable infant LAZ effects; no universal large LAZ gain
5	Ashorn et al. [16]	AJCN/Matern Child Nutr (iLiNS-DYAD Malawi)	Malawi	Randomized controlled trial	Pregnant women → infants	Maternal SQ-LNS in pregnancy (±lactation)	Iron-folic acid/standard care	No clear increase in mean birth size; downstream infant LAZ effects mixed and modest
6	Maleta/Mangani et al. [17, 18]	AJCN/ Matern Child Nutr	Malawi	Randomized trial (infant LNS)	Infants 6–18 months	Child LNS (milk-based vs. soy LNS)	CSB++/standard complementary foods	Overall LAZ effect small; some subgroup improvements and reductions in severe stunting in certain arms
7	Phuka et al. ([19]/follow-ups)	Public Health Nutrition/J Nutr	Malawi	Randomized controlled trial	Young children 6–24 months	Ready-to-use fortified spreads (RUSF/FS)	Control/biscuits/CSB	Lower incidence of severe stunting in fortified-spread groups vs. control at site
8	Lanou et al. [20]	BMC/Nutrition journals	Burkina Faso	Cluster-randomized controlled trial	Children 6–23 months	Micronutrient powders (MNP) + nutrition education (home fortification)	Usual care/no MNP	Marginal growth effects: no significant mean LAZ change overall; small increases in growth velocity in nested analyses; subgroup signals
9	Christian et al. (JiVitA series)	Lancet/AJCN (trial reports)	Bangladesh	Randomized controlled trial (maternal MMN)	Pregnant women → offspring	Maternal multiple micronutrient (MMN) supplementation in pregnancy	Iron-folic acid	Early improvements in infant length in some analyses; sustained effect on stunting by 24 months often not observed (attenuation over time)
10	Rang-Din nutrition study/RDNS (Dewey et al. trials)	FHI reports/AJCN follow-ups	Bangladesh	Randomized trial (maternal & child LNS arms)	Pregnant women, infants 6–24 months	Lipid-based nutrient supplements (maternal/child)	Usual care/placebo	Short-term/subgroup benefits; long-term sustained HAZ gains limited except in subgroups
11	Various RUSF/fortified complementary food RCTs	AJCN/Public health nutrition etc.	Multiple (food-insecure settings)	Randomized controlled trials	Children 6–24/36 months	Fortified complementary foods (RUSF, CSB++, fortified blends)	Other supplements/standard diet	Weight gains consistent; HAZ effects small–modest; some trials show reduced stunting in high-need populations
12	Soofi et al. [21]	American J Clin Nutr (2024)	Afghanistan	Quasi-experimental (community pre–post with control districts)	Pregnant & lactating women and children <2 years (program scale)	Specialized nutritious foods (super cereal for mothers) + MQ-LNS for children 6–23 months + SBCC	Control districts (standard care)	Stunting prevalence ↓ 5% (95% CI −9.9 to −0.2; p = 0.041); underweight ↓ 4.6%. Program improved IYCF practices
13	Iannotti et al. [22] Lulun II follow-up	Maternal & Child Nutrition (2020)	Ecuador (follow-up)	Cohort follow-up of original RCT	Children ≈3 years (Lulun follow-up)	No ongoing intervention (observational)	—	Original egg effect on LAZ not present after ∼2 years; high stunting prevalence (∼50%); current egg intake associated with reduced HAZ decline. (Demonstrates need for sustained support)

### Risk of Bias

Risk of bias findings are summarized in Table 2. Overall, most randomized controlled trials were judged to have low risk of bias across key domains. Some concerns were primarily related to blinding, given the practical limitations inherent in food-based interventions. The quasi-experimental and cohort follow-up studies were assessed as having moderate risk of bias, mainly due to potential confounding and attrition.

**TABLE 2:** Risk of bias assessment of included primary studies (Asia, Africa, and Latin America, 2008–2024).

Study	Study design	Randomization process	Allocation concealment	Blinding of outcome assessment	Incomplete outcome data	Selective reporting	Overall risk
Iannotti [12]	RCT	Low	Low	Some concerns	Low	Low	Low
Adu-Afarwuah [15]	RCT	Low	Low	Some concerns	Low	Low	Low
Ashorn [16]	RCT	Low	Low	Some concerns	Low	Low	Low
Maleta [18]	RCT	Low	Low	Some concerns	Low	Low	Low
Phuka [19]	RCT	Low	Low	Some concerns	Low	Low	Low
Lanou [20]	Cluster RCT	Low	Some concerns	High	Low	Low	Moderate
Christian (JiVitA)	RCT	Low	Low	Some concerns	Low	Low	Low
RDNS	RCT	Low	Low	Some concerns	Low	Low	Low
Soofi [21]	Quasi-experimental	Moderate	Not applicable	High	Moderate	Low	Moderate
Lulun II 2020	Cohort follow-up	Not applicable	Not applicable	Not applicable	Moderate	Low	Moderate

### Effects of Nutrition-Specific Interventions on Linear Growth and Stunting

#### Lipid-Based Nutrient Supplements (LNS)

Small-quantity lipid-based nutrient supplements were evaluated in multiple randomized controlled trials and pooled analyses. An individual participant data meta-analysis reported a mean difference in length-for-age z-score (LAZ) of approximately +0.14 and a relative reduction in stunting prevalence of approximately 12% compared with control groups. Randomized trials conducted in Ghana and Malawi also reported modest improvements in linear growth outcomes among children receiving LNS, with effect sizes varying across study settings and populations [13, 15, 16].

### Micronutrient Supplementation and Home Fortification

Micronutrient powders and maternal multiple micronutrient supplementation were assessed in cluster-randomized and randomized trials conducted in Burkina Faso and Bangladesh. These studies reported no statistically significant differences in mean LAZ between intervention and control groups, although small improvements in growth velocity were observed in specific age subgroups. Prenatal MMN supplementation trials documented early improvements in infant length, with limited effects on stunting prevalence observed at later follow-up periods [20, 23].

### Fortified Complementary Foods and Ready-to-Use Products

Several randomized trials evaluated fortified complementary foods and ready-to-use supplementary foods. Studies conducted in Malawi reported small but statistically significant improvements in height-for-age z-scores and reductions in severe stunting among children receiving fortified spreads compared with corn–soy blends or standard diets [18, 19]. Additional trials conducted in food-insecure settings reported consistent improvements in weight-related indicators, with modest effects on linear growth outcomes [24].

### Food-Based Interventions

A randomized controlled trial conducted in Ecuador evaluated the effect of daily egg supplementation during infancy. Children who received one egg per day for 6 months demonstrated an increase of approximately +0.63 in length-for-age z-scores and a reduction in stunting prevalence of approximately 47% compared with controls at endline. Follow-up assessments indicated attenuation of the initial growth effects over time [12].

### Integrated Community-Based Programs

A quasi-experimental program conducted in Afghanistan combining specialized food supplementation with social and behavior change communication reported a reduction in stunting prevalence of approximately 5% and a reduction in underweight prevalence of 4.6% compared with control districts [21].

Across the included studies, nutrition-specific interventions were associated with improvements in child linear growth outcomes of varying magnitude. Reported effect sizes ranged from small increases in LAZ in supplementation-based trials to larger reductions in stunting prevalence in food-based and multi-component intervention programs.

The certainty of evidence for primary growth outcomes was evaluated in accordance with GRADE principles and is summarized in Table 3. Evidence supporting improvements in length-for-age z-scores and reductions in stunting prevalence was rated as moderate certainty. Downgrading was primarily due to heterogeneity in intervention design, variability in contextual factors, and limitations related to blinding. Evidence related to micronutrient powder interventions was rated as low certainty due to inconsistency across studies and imprecision of effect estimates.

**TABLE 3:** Summary of findings and certainty of evidence for nutrition-specific interventions on child growth outcomes (Asia, Africa, and Latin America, 2008–2024).

Outcome	No. of studies	Direction of effect	Magnitude of effect	Certainty of evidence
Improvement in LAZ	9	Positive	Small to moderate	Moderate
Reduction in stunting prevalence	7	Positive	Small to moderate	Moderate
Micronutrient powders on growth	4	Inconsistent	Minimal	Low
Food-based interventions	3	Positive	Moderate to large (short-term)	Moderate
Integrated community programs	2	Positive	Modest	Moderate

## Discussion

### Interpretation and Comparison With Previous Studies

The overall pattern of findings from this systematic review aligns with evidence from broader global syntheses indicating that nutrition-specific interventions can contribute to reductions in child stunting, particularly when implemented during the first 1,000 days of life. A comprehensive package of proven interventions including micronutrient supplementation, breastfeeding promotion, complementary feeding, and management of acute malnutrition has been estimated to prevent approximately one-fifth of stunting cases worldwide [25]. The results of the present review are consistent with this estimate, demonstrating measurable, though context-dependent, improvements in child linear growth across diverse low- and middle-income settings.

Evidence from randomized trials and pooled analyses indicates that lipid-based nutrient supplements are associated with modest but meaningful improvements in length-for-age z-scores and reductions in stunting prevalence when provided during the complementary feeding period [13, 18, 26, 27]. However, the magnitude of these effects varies substantially across populations. Smaller gains are typically observed in settings with lower levels of food insecurity, underscoring the importance of contextual determinants such as baseline diet quality, infection burden, and overall living conditions in shaping intervention effectiveness.

In contrast, interventions based on micronutrient powders or multiple micronutrient supplementation have frequently demonstrated limited or inconsistent effects on linear growth [28]. While these strategies are effective in improving micronutrient status and reducing anemia, their impact on height gain is generally modest. This suggests that correction of micronutrient deficiencies alone may be insufficient to promote linear growth in environments where children continue to face recurrent infections, inadequate energy intake, or suboptimal caregiving conditions.

Stronger growth effects have been reported in food-based interventions. Provision of culturally acceptable, locally sourced foods has been associated with substantial short-term improvements in child linear growth, although attenuation of effects over time has been observed when interventions are not sustained [29]. Similar findings have been reported in programs linking household food production with behavior change communication, highlighting potential advantages in acceptability and early impact when food-based strategies are embedded within local food systems [30].

Integrated community-based nutrition programs combining food supplementation with social and behavior change communication have also demonstrated measurable reductions in stunting prevalence at the population level [25, 31]. These findings emphasize that improvements in caregiver knowledge, feeding practices, and household behaviors are critical complements to nutrition-specific inputs and are likely to enhance both uptake and effectiveness of interventions.

### Biological and Contextual Mechanisms

The biological plausibility underlying these findings is well established. Improved energy and micronutrient intake supports key endocrine pathways, including insulin-like growth factor-1 and thyroid hormones, which regulate bone and tissue growth, while also enhancing immune function and reducing susceptibility to infection [28, 32]. Adequate nutrition during early life therefore provides the physiological foundation necessary for normal linear growth.

However, these biological benefits are contingent upon supportive environmental conditions. Repeated exposure to enteric pathogens, poor sanitation, and chronic inflammation can blunt growth responses even in the presence of improved diets. Evidence from trials integrating nutrition with water, sanitation, and hygiene interventions suggests that combined approaches yield greater improvements in linear growth than either intervention alone [28, 32]. Social and household factors including maternal education, poverty, and household food diversity also play a critical role in determining whether nutritional interventions translate into sustained growth gains [30]. Together, these findings highlight the dynamic interaction between biological processes and environmental exposures in shaping child growth trajectories.

### Strengths and Limitations

This review has several strengths. It synthesizes evidence from randomized and quasi-experimental studies conducted across multiple regions, offering a comprehensive perspective on the effectiveness of nutrition-specific interventions in diverse contexts. The focus on the first 1,000 days of life and the inclusion of both supplementation-based and food-based strategies enhance the relevance of the findings for policy and program design.

Nevertheless, important limitations should be acknowledged. Substantial heterogeneity across studies in terms of intervention type, dosage, duration, outcome definitions, and follow-up periods precluded quantitative meta-analysis and limits direct comparability of effect estimates. Long-term follow-up data were limited in many trials, restricting assessment of the sustainability of observed growth effects. In addition, the frequent implementation of nutrition-specific interventions alongside broader community or WASH activities complicates attribution of observed outcomes to nutrition-specific components alone.

### Methodological Quality and Certainty of Evidence

Interpretation of these findings should also consider the methodological quality of the included studies. Most randomized trials were assessed as having low overall risk of bias, although blinding was frequently limited due to the practical realities of food-based interventions. While such limitations are common in nutrition research, they may introduce modest performance or detection biases.

The overall certainty of evidence for improvements in linear growth and reductions in stunting was judged to be moderate. This indicates reasonable confidence in the direction of observed effects, although effect magnitude may vary according to contextual factors such as baseline nutritional vulnerability, implementation fidelity, and infection burden. Importantly, the evidence supports consistent but generally modest improvements rather than large or universal impacts.

By contrast, evidence regarding micronutrient powder interventions was characterized by lower certainty, primarily due to inconsistency across settings. This variability reinforces the understanding that correction of micronutrient deficiencies alone may not be sufficient to generate measurable gains in linear growth without concurrent improvements in dietary adequacy and environmental health conditions.

In interpreting these findings, it is important to consider study-level characteristics that may influence effect estimates. Sample sizes varied substantially across included trials, ranging from small single-site RCTs to large multi-country analyses. Smaller studies occasionally reported larger effect sizes, which may reflect contextual intensity or limited precision, whereas larger pooled analyses demonstrated more modest but statistically robust estimates.

Similarly, intervention duration differed considerably across studies. Short-term interventions often produced measurable improvements in linear growth at endline, yet attenuation of effects was observed in follow-up assessments when supplementation was discontinued. This pattern suggests that sustained exposure may be critical for maintaining growth gains.

Baseline nutritional vulnerability also appeared to modify intervention impact. Studies conducted in populations with higher baseline stunting prevalence and food insecurity tended to demonstrate greater relative benefits, supporting the hypothesis that initial nutritional status influences responsiveness to nutrition-specific interventions.

The overall moderate certainty of evidence indicates reasonable confidence in the direction of effect, although variability in magnitude across contexts warrants cautious interpretation and limits universal generalization.

### Policy Implications and Future Directions

Overall, the evidence indicates that nutrition-specific interventions can contribute to reductions in child stunting, particularly when implemented early, sustained over time, and delivered in contexts of high nutritional vulnerability. However, the variability in effect sizes underscores that such interventions are most effective when embedded within broader strategies addressing infection control, sanitation, caregiver practices, and socioeconomic constraints [25, 30, 31, 33–36]. This is consistent with previous evidence indicating that micronutrient supplementation alone has limited impact on linear growth, while integrated and context-sensitive approaches are required to achieve meaningful and sustained improvements in child development outcomes [34–36]. Future research should prioritize long-term follow-up, improved assessment of contextual modifiers, and rigorous evaluation of integrated approaches that combine nutrition with complementary health and social interventions.

In summary, nutrition-specific interventions implemented during the first 1,000 days are associated with consistent but modest improvements in child linear growth. The moderate certainty of evidence supports cautious confidence in these effects while underscoring the importance of context-sensitive implementation and continued methodological rigor in future trials. Sustainable progress in stunting reduction will likely depend on integrating nutrition-specific actions within broader structural and environmental reforms.
